# Role of insulin as a growth promoter in regulating the response of curcumin in human primary gingival fibroblasts: An *in vitro* study

**DOI:** 10.4103/0972-124X.60225

**Published:** 2009

**Authors:** Jaya Dixit, Umesh Verma, Karamjeet, Ramesh Sharma, Anil K. Balapure

**Affiliations:** *Dental Faculty; CSMMU (erstwhile KGMC), Lucknow, India*; 1 *Department of Periodontics, CSMMU (erstwhile KGMC), Lucknow, India*; 2 *CDRI, Lucknow, India*

**Keywords:** Apoptosis, curcumin, cytotoxicity, gingival fibroblasts, insulin

## Abstract

**Background:**

The aim of this investigation was to evaluate the biochemical and morphologic changes in human primary gingival fibroblasts (hPGF) treated with curcumin (CUR) and insulin (I) plus curcumin (CUR) in a dose-dependent fashion.

**Materials and Methods:**

Human gingival fibroblasts were obtained from gingival biopsies. Curcumin was dissolved in ethanol, diluted with Dulbecco's modified Eagle's medium (DMEM) to obtain dilutions and bovine insulin was dissolved in 0.01 N HCl and diluted with DMEM. Cells were exposed to different concentrations of CUR and insulin (1 μg/ml) plus CUR for next 48 hours at 37°C and cellular growth profile was assessed using sulforhodamine-B (SRB), 3-(4,5-dimethylthiazol-2-yl)-2,5-diphenyltetrazolium bromide (MTT) and fluorescence-activated cell sorter (FACS).

**Results:**

The cell viability in both the treatments at lower concentrations of SRB (1 and 10 μM) and MTT (1 μM) were found to be significantly higher than that observed at higher concentrations, while apoptosis in both the treatments at lower concentrations was observed to be significantly lower than at higher concentrations. Also, the cell viability of I + CUR at lower concentrations of SRB (1, 10 and 25 μM) and MTT (1 μM) were found to be significantly higher than the respective CUR, while apoptosis at higher concentrations (50, 75 and 100 μM), especially at 75 μM was significantly low. The IC_50_ of I + CUR of SRB, MTT and FACS were 1.1, 1.0 and 1.4 times higher than respective concentrations of CUR.

**Conclusions:**

Insulin (1 μg/ml) exerted cytoproliferative and curcumin exerted cytocidal effects (in a dose-dependent manner) on hPGF. Insulin (1 μg/ml) and curcumin at different concentrations when added together decreased the cytocidal effect of curcumin.

## INTRODUCTION

The major function of insulin is to counter the concerted action(s) of a number of hyperglycemia-generating hormones and to maintain low blood glucose levels. It also stimulates lipogenesis; diminishes lipolysis; increases amino acid transport into cells; modulates transcription; alters cell contents of numerous mRNAs; stimulates growth, DNA synthesis, cell replication; and has effects identical to insulin-like growth factors (IGFs) and relaxin. Insulin also possesses well-known activities in controlling energy metabolism, cellular proliferation and biosynthesis of functional molecules to maintain a biological homeostasis. Recently, several studies have suggested that insulin may protect cells from apoptosis in different cell lines; however, little is known about the nature of its anti-apoptotic activity.[1] Studies have also demonstrated that IGF-I has mitogenic effect(s) on fibroblasts originating from various connective tissue and cell lines.[2] In animal studies, increase in the DNA content of human gingival fibroblasts (hGF) has been directly linked to the increased insulin levels.[3] Insulin also causes reversal of impaired fibroblast functions caused by hyperglycemia.[4]

Besides insulin, one of the plant derived medicines such as curcumin has a long history for treatments in medicines due to its plethora of beneficial effects. Its anti-infective spectrum includes antibacterial,[5] antiviral, antifungal, antimalarial, nematocidal, antiprotozoal and antivenom[6] properties.

A hypothesis investigated that a direct stimulatory action on pancreatic-*β* cells [SS1]could contribute toward the hypoglycemic activity of this compound. Animal studies also have shown that curcumin administration resulted in an increase in amounts of transforming growth factor (TGF)-*β*_1_. TGF-*β*_1_ stimulates fibroblast division at low concentration and stimulates differentiation at high concentration. The aim of this investigation was to evaluate the biochemical and morphologic changes in human primary gingival fibroblasts (hPGF) treated with curcumin and insulin plus curcumin in a dose-dependent fashion.

## MATERIALS AND METHODS

The curcumin (CUR) and bovine insulin (I) used for this experimental study were procured from Sigma Chemical Co., St. Louis, MO (USA). Along with these, all reagents used for culture of human gingival fibroblasts were also procured from Sigma Chemical Co., St. Louis, MO (USA). Sterile plasticware, for example, Flasks, 6-/12-/96-well tissue culture plates were obtained from Corning, Boston, MA (USA).

Human gingival fibroblasts used in this study were obtained from gingival biopsies, under aseptic conditions, with informed consent from six patients and were transferred on ice to the Tissue and Cell Culture Unit (TCCU) at Central Drug Research Institute (CDRI), Lucknow. Curcumin was dissolved in ethanol, diluted with Dulbecco's modified Eagle's minimal essential medium (DMEM) to obtain dilutions and bovine insulin was dissolved in 0.01 N HCl and diluted with DMEM.

For experiments of insulin, curcumin and insulin plus curcumin vis-a-vis fibroblasts, the cells were harvested after trypsination. A total of 0.1 × 10^6^ cells were plated in 12-well plates in DMEM with 0.2% fetal bovine serum (FBS) and incubated for 24 hours at 37°C in a humidified CO_2_ incubator. In our pilot study, SRB assay showed that hPGF cells optimally proliferated in 0.2% FBS in comparison with FBS-free condition; thus, in each experiment it was considered as control. In the same pilot study when hPGF cells were exposed to various concentrations of insulin under 0.2% FBS conditions for 48 hours, the effects were analyzed by MTT, SRB and flow cytometry analysis. These analyses revealed that insulin has statistically significant cytoproliferative effect at 1 μg/ml. Subsequently, cells were exposed to different concentrations of curcumin (1, 10, 25, 50, 60, 75, 100 μM) and insulin (1 μg/ml) plus curcumin for the next 48 hours at 37°C. After treatment, the cells were washed thrice with chilled medium and then further incubated at 37°C in a humidified 5% CO_2_ incubator for the next 48 hours. At the end of the experiment, the cells were photographed using a Nikon Phase-Contrast Microscope with a Nikon Digital Camera (Coolpix 5400). The cells were harvested from a confluent flask using 0.1% Trypsin-EDTA solution in Ca^++^ and Mg^++^ free PBS and plated in 96-well microtiter plate (1 × 10^4^ cells/well) for conducting the cell viability assays MTT and SRB as well as flow cytometry. After 24 hours of culture, the cells were treated with various ligands for appropriate duration, then washed with medium and further cultured for next 48 hours.

The MTT[7] ([3-(4,5-dimethylthiazol-2-yl)- 2,5- dipheny ltetrazolium bromide]), SRB[7,8] (Sulforhodamine B) and Flourescence Activated Cell Sorter (FACS) or flow cytometry[9] analyses were performed to assess cellular growth profile. Effects of various concentrations of curcumin and insulin plus curcumin were evaluated.

### Statistical analysis

Data were analyzed using two-factor (drugs × concentrations) analysis of variance (ANOVA) with (SRB and MTT assays) and without (FACS assay) replications, and the significance of mean difference within factor was carried out with the Newman-Keuls post hoc test. Association between response (cell viability and apoptosis) and concentrations was calculated by Pearson correlation coefficient (r). Probit analysis was used to calculate IC_50_. A two-tailed (α = 2) probability (*P*) value less than 0.05 (*P* < 0.05) was considered to be statistically significant. For analyses, MS EXCEL (MS Office 97-2003), GraphPad Prism (Demo, Version 5) and STATISTICA (version 7) software were used.

## RESULTS

**Figure 1:**
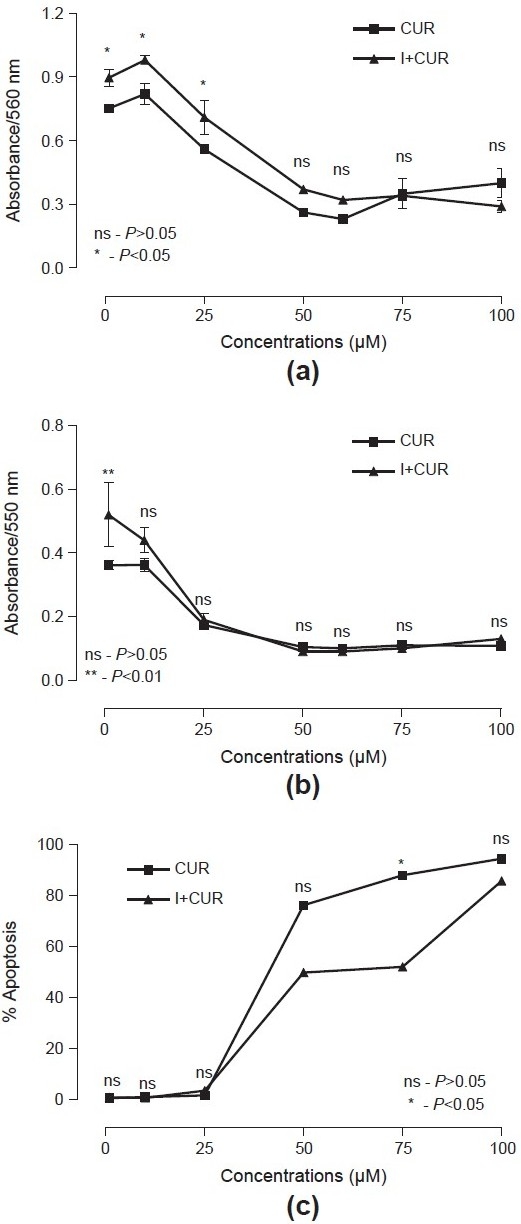
Mean (± SE) responses of curcumin and I + curcumin in sulforhodamine-B; (a) MTT; (b) and fluorescence activated cell sorter; (c) assays at different concentrations and significance of mean difference between drugs for each concentration

Cell viability (SRB and MTT assays) and apoptosis (FACS assay) of two drug treatments (CUR and I + CUR) at different concentrations of respective assays are summarized in Table 1, and the significance of mean difference between treatments of three assays in each concentration is shown graphically in Figure 1 and Table 1. Figure 1 shows that the cell viability in both the treatments of SRB and MTT assays follows the same trend, that is, decreases with increasing concentrations. The cell viability is high in SRB than MTT and higher in I + CUR than CUR. Similarly, apoptosis in both the treatments increases with increasing concentrations and apoptosis is higher in CUR than I + CUR. Comparing mean between concentrations for each treatment, the cell viability in both treatments at lower concentrations of SRB (1 and 10 μM) and MTT (1 μM) was found to be significantly (*P* < 0.01) high than that the higher concentrations (25, 50, 60, 75 and 100 μM), while apoptosis in both the treatments at lower concentrations (1, 10, 25 μM) was significantly (*P* < 0.01) lower than the higher (50, 75 and 100 μM) concentrations. Comparing mean between treatments for each concentration, the cell viability of I + CUR at lower concentrations of SRB (1, 10, 25 μM) and MTT (1 μM) were found to be significantly (*P* < 0.05) higher than the respective CUR, while apoptosis at higher concentrations (50, 75, 100 μM), especially at 75 μM is significantly (*P* < 0.05) lower [Figure 1]. The correlation coefficient (*r*) of cell viability (SRB and MTT assays) and apoptosis (FACS assay) with concentrations and between cell viability and apoptosis are summarized in Table 2. Table 2 shows that in both the treatments, cell viability had significant (*P* < 0.01) inverse relation (negative correlation) with concentration while direct relation (positive correlation) with apoptosis. In SRB, as concentrations increases, cell viability decreases more in CUR (*r* = −0.76) than in I + CUR (*r* = −0.92), while in MTT it was found to be similar between CUR (*r* = −0.83) and I + CUR (*r* = −0.81). Similarly, with increasing concentrations, apoptosis in CUR (*r* = 0.94) and I + CUR (*r* = 0.97) was also found to be similar. The percent inhibition (IC_50_) of cell viability (SRB and MTT assays) and apoptosis (FACS assay) in the two treatments of respective assays are summarized in Table 3. The IC_50_ for CUR by SRB, MTT and FACS were found to be 58.69, 39.67 and 43.71, respectively, as compared with higher values of 65.46, 40.55 and 59.68, respectively, for the I + CUR group.

**Table 1:** Statistics (Mean ± SE) of drugs (curcumin and I + curcumin) responses in sulforhodamine-B (absorbance/560 nm), MTT (absorbance/550 nm) and fluorescence activated cell sorter (% apoptosis) assays at different concentrations (μM)

Conc.	SRB		MTT		FACS	
			
	CUR (n = 3)	I + CUR (n = 3)	CUR (n = 3)	I + CUR (n = 3)	CUR (n = 1)	I + CUR (n = 1)
1	0.75 ± 0.01	0.90 ± 0.04	0.36 ± 0.01	0.52 ± 0.10	0.55	0.81
10	0.82 ± 0.05	0.98 ± 0.02	0.36 ± 0.02	0.44 ± 0.04	0.89	0.64
25	0.56 ± 0.02	0.71 ± 0.08	0.17 ± 0.01	0.19 ± 0.02	1.51	3.34
50	0.26 ± 0.02	0.37 ± 0.01	0.10 ± 0.00	0.09 ± 0.01	76.25	49.76
60	0.23 ± 0.01	0.32 ± 0.01	0.10 ± 0.01	0.09 ± 0.01	-	-
75	0.35 ± 0.07	0.34 ± 0.02	0.11 ± 0.01	0.10 ± 0.00	88.03	51.98
100	0.40 ± 0.07	0.29 ± 0.03	0.11 ± 0.01	0.13 ± 0.01	94.47	85.76

**Table 2:** Correlation (n = 7 or n = 6) between drugs (curcumin and I + curcumin), responses (absorbance and apoptosis) and concentrations and between absorbance (sulforhodamine-B and MTT) and apoptosis (fluorescence activated cell sorter)

Variables		SRB		MTT		FACS	
				
	Conc.	CUR	I + CUR	CUR	I + CUR	CUR	I + CUR
Conc.	1.00						
SRB (CUR)	−0.76**	1.00					
SRB (I + CUR)	−0.92**	0.95**	1.00				
MTT (CUR)	−0.83**	0.95**	0.95**	1.00			
MTT (I + CUR)	−0.81**	0.94**	0.92**	0.99**	1.00		
FACS (CUR)	0.94**	−0.88**	−0.96**	−0.83**	−0.80**	1.00	
FACS (I + CUR)	0.97**	−0.80**	−0.93**	−0.79**	−0.75**	0.96**	1.00

** −*P*, < 0.01

**Table 3:** Estimated IC_50_ (μM) with 95% lower and upper confidence interval of two drugs of three assays

Assays	CUR			I + CUR			Ratio IC_50_		
			
	IC_50_	Lower	Upper	IC_50_	Lower	Upper	(I + CUR/CUR)
SRB	58.69	51.35	67.08	65.46	59.86	71.60	1.1
MTT	39.67	35.05	44.90	40.55	35.90	45.80	1.0
FACS	43.71	40.64	47.02	59.68	54.97	64.79	1.4

**Figure 2:**
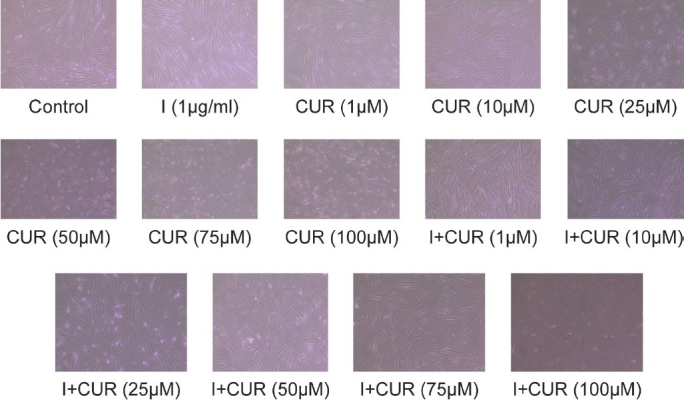
Cytomorphological analysis of curcumin- and I + curcumin-treated human primary gingival fibroblasts cells: 0.2 × 10^6^ cells were precultured for 24 hours in 0.2% fetal bovine serum-supplemented Dulbecco's modified Eagle's minimal essential medium and then exposed to various concentrations of curcumin and I + curcumin for 48 hours. Subsequently, cells were observed and photographed under inverted phase-contrast Nikon microscope (magnification ×100). All pictures are typical of three independent experiments, each performed under identical conditions

Cellular morphology: Evidently, the hPGF cells appeared healthy and abundant significantly in its growth phase when exposed to 0.2% FBS. About 1 μg/ml insulin augmented the cell growth maximally and significantly. It exerted cytoproliferative response. When hPGF cells were exposed to different concentrations of CUR it exerted dose-dependent cytocidal effect and cells started shrinking in size, with maximum cytotoxicity at a concentration of 75 μM [Figure 2]. Upon exposure to different concentrations of I + CUR, the cells expressed less shrinkage as compared with exposure to the same concentration of CUR. Their population was also greater as compared with exposure to the same concentration of CUR [Figure 2].

## DISCUSSION

In this study, we investigated the role of insulin (I) as a mitogen in augmenting the cytostatic/cytoproliferative/cytocidal effect of curcumin (CUR), on in-house developed and cultured normal hPGF. The hPGF cells were used to titrate the minimal and optimal fetal bovine serum (0.2% FBS) concentrations through SRB assay to minimize the endogenous peptides besides insulin. Zebroswki *et al.* reported that serum factors interfere with the availability of insulin in the cells.[3]

Insulin at a concentration of 1 μg/ml was used, as it showed statistically significant mitogenic effects on hPGF cells through a pilot study. Crace *et al.* investigated the mitogenic actions of insulin on fetal and neonatal rat cells *in vitro*. Myoblasts from fetuses showed a marked response to insulin, a significant increase over control values was observed in five of seven experiments, suggesting that insulin may have a direct role in fetal muscle growth.[10] Wallace *et al*. also reported direct mitogenic effects of insulin on normal rat prostate epithelial cells (PEC) in serum free, primary cell culture.[11]

Apoptosis of hPGF cells was low at 1, 10 and 25 μM doses of curcumin but at higher doses (50, 60, 75 and 100 μM) statistically significant high apoptosis was noted [Figure 1 and Table 1]. Through MTT assay, significant cell viability at 1 μM of curcumin was observed [Figure 1 and Table 1]. In context to our results of curcumin on hPGF, Kostandova and Pamula analyzed the effects of curcumin-treated normal human fibroblasts and micro-vascular endothelial cells (hMVEC) using MTT assay and observed that lower doses of curcumin stimulated the proliferation of normal human fibroblasts and hMVEC, whereas higher doses inhibited it.[12] Gaedke *et al*., in their experiments on renal fibroblasts and mesangial cells, using MTT assay reported that curcumin was nontoxic at doses of 0 to 10 mM/L.[13] Kim *et al.* observed no cytotoxicity in treatment with 15 mM curcumin on human umbilical vein endothelial cells (HUVEC) using MTT assay.[14] Curcumin-treated hPGF cells exhibited maximum and significant apoptosis at 75 μM showing decrease in cell population and shrinkage of cell size, when compared with 0.2% FBS-treated cells [Figure 2]. Jee *et al.* examined the morphologic alterations in basal cell carcinoma (BCC) cells after treatment with 50 mM curcumin and found cell shrinkage, disappearance of microvilli and appearance of membrane blebbing when compared with untreated cells.[15]

Intriguingly, insulin (1 μg/ml) and curcumin at different concentrations when added together decreased the cytocidal effect of curcumin [Figure 1 and Tables 1–3]. Cell viabilities at 1, 10 and 25 μM for SRB and 1 μM for MTT assay were significantly higher than curcumin alone. Also, apoptosis of cells at 50, 75 and 100 μM concentrations was significantly low as compared with curcumin alone. Statistically significant high apoptosis (88.03%) at 75 μM of curcumin was reduced to 51.98% when combined with 1 μg/ml of insulin, that is, reduction of 36.05% apoptosis; however, on further increasing dose of curcumin to 100 μM reduction in apoptosis was 8.71%. This could be explained in that higher concentrations (100 μM) of curcumin caused a very high apoptosis of 94.47%; however, insulin decreased its apoptotic effect to 85.76%, but it was not statistically significant. These findings were supported by cellular morphology revealing augmentation of cell population and decrease in cell shrinkage. Increase in antiapoptotic activity of insulin was further observed, when IC50 for I + CUR in SRB, MTT and FACS (apoptosis) assays were 1.1, 1.0 and 1.4 times, respectively, higher than the respective CUR alone [Table 3]. This might depict the possible antiapoptotic and mitogenic actions of insulin; however, the exact mechanism is unknown. Kang *et al.* observed that Insulin exerted antiapoptotic activity by suppression of reactive oxygen species in HepG_2_ (hepatoblastonema cell line), which expresses intrinsic insulin receptors.[1] Augustin *et al.* (2003) reported that insulin in addition to the regulation of glucose transport, exerts mitogenic and antiapoptotic activities.[16]

This *in vitro* investigation has come up with an outcome that combination doses of insulin (1 μg/ml) + CUR (1 μM) are optimal, which proliferates maximum in both the SRB and MTT assays. To our knowledge, till date no such studies have been reported, which evaluated synergistic/antagonistic effects of combinations of insulin and curcumin on hPGF cells.

Curcumin (1%) as subgingival irrigant resulted in significant reduction in bleeding on probing (100%) and redness (96%), when compared with chlorhexidine and saline group as an adjunctive therapy in periodontitis patients.[17] Because curcumin-induced apoptosis is decreased by insulin as shown in the present study, curcumin as subgingival irrigant will be a novel approach for diabetic patients (on insulin therapy) in the treatment of periodontitis. However, further studies are required to substantiate and correlate these findings in clinical situations and explore the exact mechanism for curcumin-induced apoptosis of hPGF cells and its decrease in apoptotic effect by insulin.
